# A novel instrument for ligament balancing: a biomechanical study in human cadaveric knees

**DOI:** 10.1186/s40634-023-00643-7

**Published:** 2023-08-16

**Authors:** Lars H. W. Engseth, Jørgen Grønsund, Eirik Aunan, Jan Egil Brattgjerd, Anselm Schulz, Gilbert Moatshe, Stephan M. Röhrl

**Affiliations:** 1 Department of Orthopaedics, Oslo University Hospital Ullevål (OUH), P.O Box 4956, Nydalen, Oslo, 0424 Norway; 2 Department of Surgery, Innlandet Hospital Trust, Lillehammer, Norway; 3 Faculty of Medicine, University of Oslo, Oslo, Norway; 4 Center for Implant and Related Research Oslo (CIRRO), Oslo, Norway; 5 CapNor, Sola, Norway; 6 Faculty of Health Sciences, OsloMet, Oslo, Norway; 7 Department of Radiology and Nuclear Medicine, Oslo University Hospital Ullevål, Oslo, Norway

**Keywords:** Pie-crusting, Balancing, Soft tissue balance, Medial collateral ligament, MCL, Bellemans, Whiteside, Ligament lengthening, Knee arthroplasty, TKA

## Abstract

**Purpose:**

Ligament balancing is a prerequisite for good function and survival in total knee arthroplasty (TKA). Various balancing techniques exist, but none have shown superior results. The pie-crusting technique by Bellemans of the medial collateral ligament is commonly utilized; however, it can be difficult to achieve repeatable ligament lengthening with this technique. Therefore, we invented a novel instrument to standardize the pie-crusting technique of the superficial and deep medial collateral ligament (hereafter MCL). The purpose was to examine if pie-crusting with the instrument could produce repeatable ligament lengthening.

**Methods:**

The MCL was isolated in 16 human cadaveric knees, and subjected to axial tension. The instrument was composed of a specific grid of holes in rows, used to guide sequential pie-crusting puncturing of the MCL with a Ø1.6 mm end-cutting cannula. Ligament lengthening was measured after each row of punctures. Regression analysis was performed on the results.

**Results:**

Mean lengthening ± SD in human cadaveric MCL for puncturing of row 1 in the instrument was 0.06 ± 0.09 mm, 0.06 ± 0.04 mm for row 2, 0.09 ± 0.08 mm for row 3, 0.06 ± 0.05 mm for row 4 and 0.06 ± 0.04 mm for row 5, giving a mean total lengthening of 0.33 ± 0.20 mm. Linear regression revealed that MCLs were repeatably lengthened by 0.07 mm per row when punctured using the instrument.

**Conclusions:**

MCLs showed linear lengthening in human cadavers for subsequent use of the instrument. Our instrument shows promising results for repeatable ligament lengthening.

## Background

Varus deformity is the most common deformity (60–80%) [1] in patients undergoing total knee arthroplasty (TKA) [2–5]. In varus knees, there could be shortening of medial structures [4]; therefore, if mechanical alignment is the goal, perpendicular bone cuts could produce a trapezoidal gap between the femur and tibia, with a shorter medial side. This imbalance should be corrected through ligament balancing as it is seen as a prerequisite for good function and survival [2–4]. Aunan et al. [2] found ligament balancing to be necessary in 70 of 100 consecutive TKAs.

**Fig. 1:**
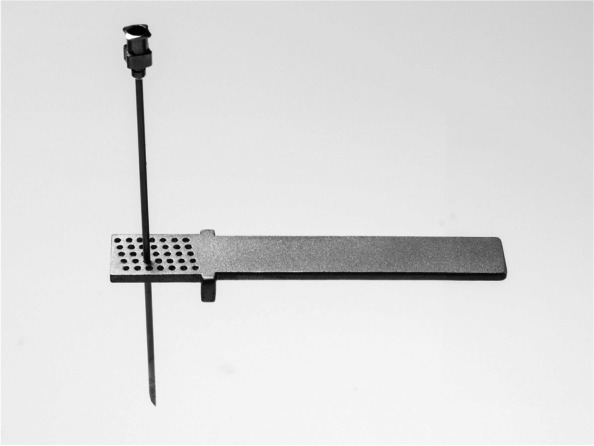
The novel instrument. Dimensions of the novel instrument for ligament balancing with the specific staggered puncturing grid. The rows are numbered 1–5 in a horizontal fashion starting from the top. Columns are numbered 1–7 from the left. An end-cutting cannula is passed through the holes to cut individual MCL-fibres

Several ligament balancing techniques exist and most focus on lengthening the soft tissue on the concave side of the knee, and therefore in varus knees Bellemans’ technique is performed with multiple perforations (pie-crusting) of the medial collateral ligament (MCL) [6], while Whiteside’s technique is performed with sequential ligament and soft tissue release, where the MCL is evaluated first. However, no technique has proved clinically superior to others [3–5, 7]. In traditional methods, it is difficult to reliably predict ligament lengthening and it relies on the performing surgeons’ feel and experience. Aunan et al. [8] found wide variation in lengthening achieved using Whiteside’s technique. Therefore, we developed a novel instrument (Fig. 1), which aspired to further develop Bellemans’ technique [3] and produce repeatable soft tissue lengthening of the MCL.

In varus knees the most important structure in ligament balancing is the superficial and deep MCL (hereafter MCL) [6]. Bellemans’ technique is a proven technique [3], which uses an end-cutting cannula to puncture the MCL by freehand, with the objective of severing some ligament fibres. When the force applied to the ligament is kept constant, each remaining fibre will be exposed to a higher force and lengthen. Bellemans’ technique lacks an objective method of guiding the puncturing, and the execution and results therefore vary. Our instrument (Fig. 1) has a specific grid that objectively guides perpendicular puncturing using an end-cutting Ø1.6 mm cannula and evenly distributes punctures throughout the ligament, which will produce a predefined spread of punctures and severing of fibres. We believe this grid is key to achieving repeatable lengthening of the MCL.

The purpose and primary outcome measure of this biomechanical study was to examine whether pie-crusting guided by the instrument could produce the same (repeatable) ligament lengthening of the MCL for several specimens. Our hypothesis was that the instrument would produce this result dependent on the number of rows punctured through the instrument and independent of ligament width. Secondary outcome measures were to determine if there were other correlations between ligament lengthening and demographic data, row-wise lengthening, cumulative number of punctures, row-wise number of punctures and load-to-failure.

## Methods

### Ethics

Human cadaveric knees were imported from ScienceCare® (USA) following regional ethical approval (REK 2018/2182 and 312,976). The same surgeon LE performed all preparations and testing.

### Instrument

The novel instrument had a horizontal five-row staggered grid consisting of seven columns with holes to accommodate end-cutting Ø1.6 mm cannulas (Fig. 1). The holes are spaced by 2.9 mm and rows were staggered with increments of 11.7° (Fig. 1). The instrument used in the study was made by 3D printing with acrylate resin. Dimensions of the grid were 20.4 mm anteroposterior and 11.6 mm craniocaudal. To guide objective perpendicular puncturing with an even spread of punctures and severing of individual fibres, the instrument should be used one row at a time.

### Preparation of human specimens

Sixteen paired knees of fresh frozen Caucasian male cadavers (Table 1) without any significant bone pathology were thawed at room temperature overnight, stripped of both menisci and all soft tissues except the MCL, and wrapped in saline-soaked gauze. An oscillating saw was used to cut the medial femoral condyle and keep the MCL with its original insertion for further testing with the 130 mm long proximal tibias.

**Table 1:** Demographic data, human cadavers

Patient	Age, years	Height, cm	Weight, kg	BMI, kg/m2
1	50	172.7	54.4	18.2
2	29	190.5	83.8	23.1
3	39	177.8	86.6	27.4
4	41	177.8	59.0	18.7
5	41	175.3	59.0	19.2
6	42	180.3	78.5	24.1
7	48	170.2	72.6	25.1
8	49	200.7	84.8	21.1
**Mean ± SD**	**42.4 ± 6.8**	**180.7 ± 9.8**	**72.3 ± 12.7**	**22.1 ± 3.2**

All specimens were males

### Testing of human specimens

**Fig. 2:**
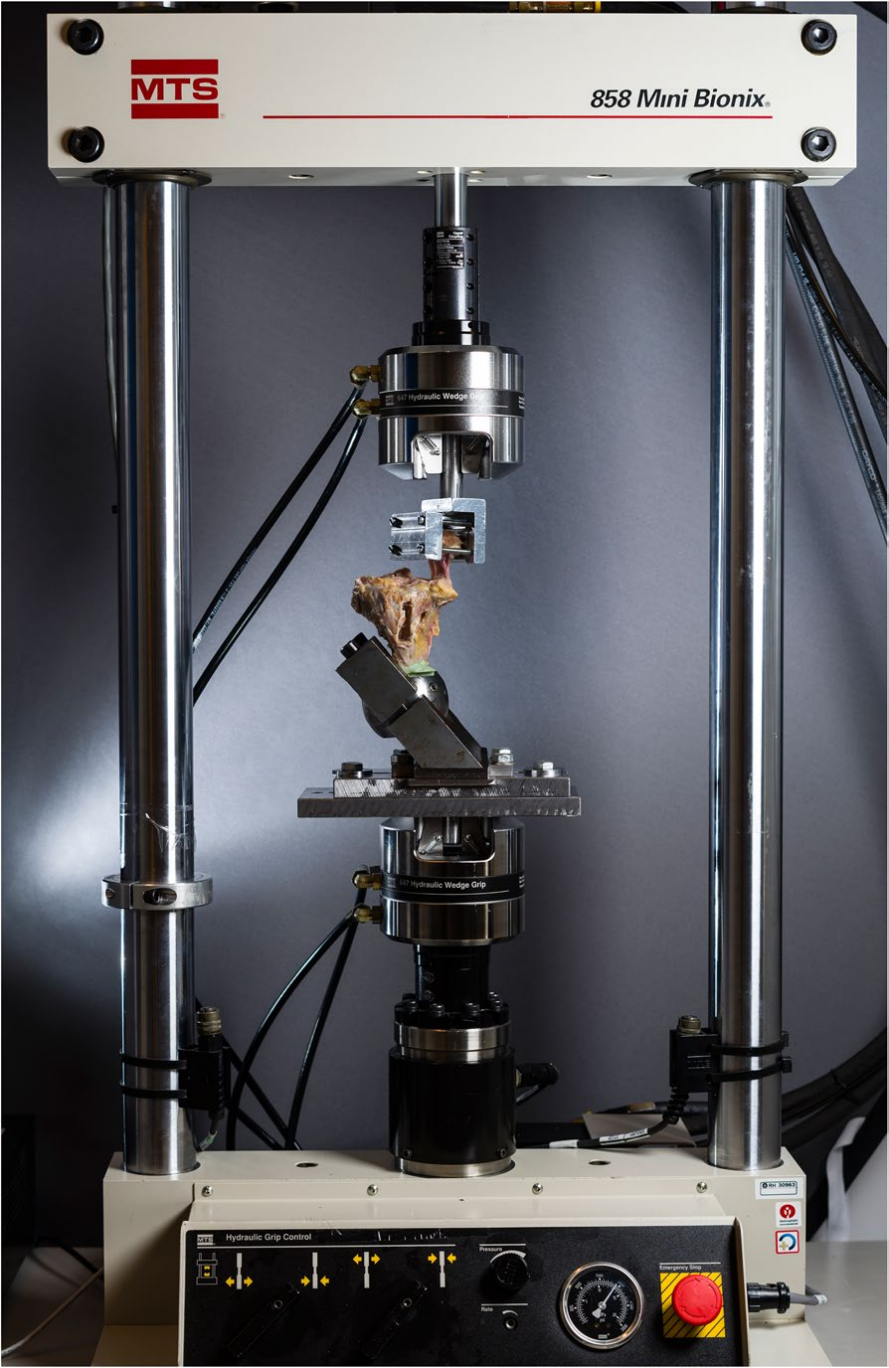
Biomechanical setup. Biomechanical setup in the MTS® machine. The tibia is fixed with polymethylmethacrylate and screws to a steel tube and the femoral bone block to a metal clamp

Proximally, the femoral bone block was trimmed and fixed in a custom-made metal clamp. For distal anchorage, the tibias with two perpendicular screws were cemented (Palacos®, Heraeus Medical, Hanau, Germany) into a steel tube. The test specimens were mounted with vertical alignment of the MCL in a hydraulic material testing machine (MiniBionix, model 858, MTS Systems®, Eden Prairie, MN, USA) (Fig. 2) with axial load cell characteristics (capacity 10 kN; resolution 1 N; displacement 1 μm; accuracy < 0.5%; sensing 5 recordings/ second). The load and displacement by the piston were recorded by a computer (MTS FlexTest 40 with Station Manager software, Eden Prairie, MN, USA).

The test specimens were subjected to preconditioning (10–50 N, 0.1 Hz, 10 cycles) and pretensioned to 10 N, before the anteroposterior MCL width at joint line was measured with digital callipers.

Following preconditioning, the specimens were subjected to a non-destructive static test. A one-minute constant tension of 10 N preceded repeated MCL length measuring and sequential puncturing using an end-cutting Ø1.6 mm cannula through the instrument until five rows were punctured. The position of the instrument at joint level was marked on the MCL.

Finally, we performed a destructive test where load was increased with an axial displacement rate of 20 mm/min. The LTF was defined as the maximum load with abrupt increments exceeding 10 mm. The mechanism of failure was inspected. When MCLs pulled out from the femur (avulsion at the femoral insertion), the remaining MCL was reinserted, and the clamp was tightened to 5 Nm. The destructive test was then restarted.

To evaluate the effect of the instrument on human MCL lengthening, we measured the MCL length after each punctured row, total lengthening after puncturing, displacement at failure and the mechanism of failure.

### Statistics

Data were analysed using Stata® 17 (StataCorp LLC, Texas, USA) and tested for normality. Descriptive statistics were expressed as mean with standard deviation (SD). To determine factors associated with ligament lengthening, regression analyses were performed to determine correlations for primary and secondary outcomes. Data showed non-parametric distribution and therefore pairwise comparison of means and Dunn’s test were used for comparisons. *P*-values < 0.05 were considered significant.

## Results

**Fig. 3:**
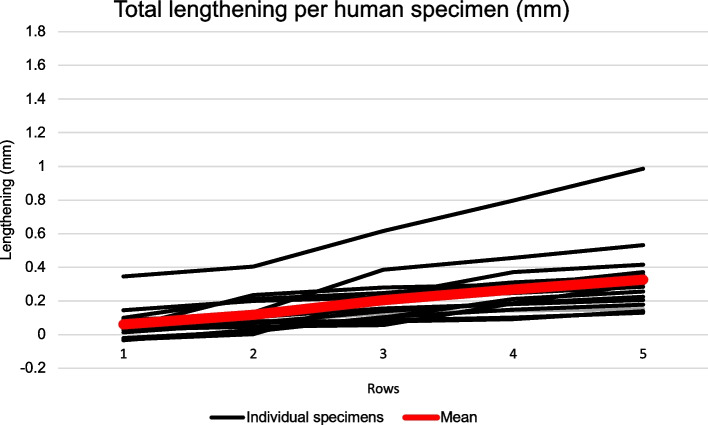
Total lengthening per human specimen. Human cadavers. Total cumulative lengthening in mm for each individual specimen (black lines) and the mean (red)

Figure 3 shows the total cumulative lengthening of the MCL with increasing number of rows punctured for each individual human specimen and the mean. There is one specimen showing higher values, but the other 15 specimens have similar lengthening patterns. Mean lengthening and number of punctures per row are listed in Table 2.

**Table 2:** MCL lengthening and number of punctures per row for human cadavers

Row	Lengthening (mean ± SD)	Punctures (mean and range)
1	0.06 ± 0.09 mm	5.3 (3–7)
2	0.06 ± 0.04 mm	5.3 (3–5)
3	0.09 ± 0.08 mm	5.6 (3–7)
4	0.06 ± 0.05 mm	5.8 (4–7)
5	0.06 ± 0.04 mm	6.0 (4–7)
**Total**	**0.33 ± 0.20 mm**	**28.0 (17–35)**

Regression analyses show that cumulative MCL lengthening can be significantly predicted by the number of rows punctured and MCL width at joint level. The regression model shows that number of rows punctured has a better predictive value than MCL width, and the predictive value is only marginally better when both predictors are combined (adjusted R^2^ of 0.40 compared to 0.30). The regression model for cumulative mean lengthening can be shown as the following linear regression equation:

- Mean lengthening row y = 0.068x – 0.009, where “y” is the total lengthening for a given row and “x” is the number of rows punctured.

The linear equation states that total lengthening was approximately 0.07 mm per row punctured. The constant (-0.009) can be ignored.

After the puncturing of row 1, subsequent lengthening is correlated with the lengthening achieved through row 1 (*p* ≤ 0.001 for all rows). Other significant relationships are seen between intrasubstance LTF and MCL width (*p* = 0.01), MCL width and lengthening per puncture (*p* = 0.002), and between lengthening per puncture and total lengthening (*p* < 0.001). Demographic data (age, body mass index (BMI), weight, height, condyle width) had no significant correlation with MCL lengthening. There were no differences between the lengthening achieved for each row (*p* = 0.87).

### Load-to-failure (LTF)

**Fig. 4:**
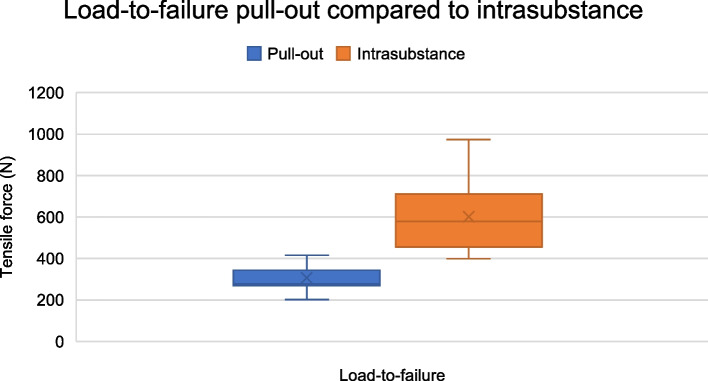
Load-to-failure. Load-to-failure of MCL intrasubstance strength compared to pull-out strength

**Fig. 5:**
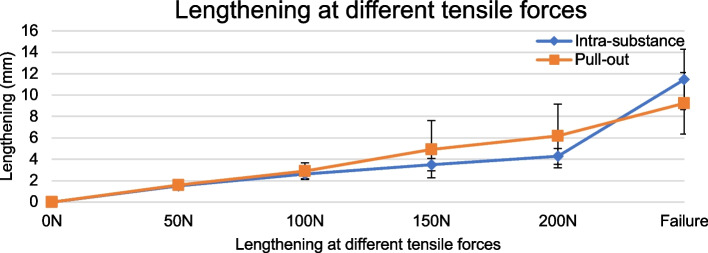
Lengthening at different tensile forces. Lengthening of MCL in mm for increasing tensile forces after puncturing was completed. Tensile strength at intrasubstance MCL failure was 527 N with an SD of 131 N, and 307.6 ± 70.2 N for pull-out failure. The lengthening is approximately linear up to 200N, as seen in the figure, but from 200N to failure the scale is not proportional and therefore cannot be used to assess linearity (Fig. 6)

Following the puncturing procedure, the MCLs were tested to failure. Nine of 16 specimens pulled away from the femoral condyle before intrasubstance failure was achieved, while the remaining seven specimens showed primary intrasubstance failure. LTF for all intrasubstance failure tests was 601 ± 165 N, but only 308 ± 70 N for primary pull-out from the femoral condyle (Fig. 4). The pull-out strength was lower than the force needed for intrasubstance failure (*p* = 0.001). After reinsertion of the nine MCLs with primary pull-out, LTF was re-examined for these ligaments. The nine specimens with primary pull-out had LTF of the MCL after reinsertion of 659 ± 171 N. The seven specimens with primary intrasubstance failure had LTF of 527 ± 131 N. Figure 5 shows how the MCL lengthened with increasing tensile force for MCLs with primary pull-out (including after reinsertion) or primary intrasubstance failure. The figure shows linear lengthening with increasing force for both groups from 50 N to failure. Lengthening between the groups did not differ significantly at any force: 50 N (*p* = 0.65), 100 N (*p* = 0.28), 150 N (*p* = 0.13), 200 N (*p* = 0.08) or at failure (*p* = 0.58). However, MCL width was correlated to intrasubstance LTF (*p* = 0.03).

### Lengthening at 170 N

In our study (Fig. 5), a tensile force of 170 N produced a mean lengthening of 3.80 ± 0.65 mm, while 10 N produced a mean total lengthening of 0.33 ± 0.20 mm. Dividing 3.80 by 0.33 gives a factor difference in lengthening of 11.5 between 10 and 170 N axial force. Lengthening per row would have increased by this factor if we had performed our testing at 170 N throughout, as MCL lengthening is linear [9]. If the results are extrapolated, lengthening per row would be approximately 0.8 mm, calculated by the previously mentioned equation:

- Mean lengthening row y = (0.068x – 0.0087) * 11.5 ≈ 0.8x, where “y” is the total lengthening for a given row, “x” is the number of rows punctured, and 11.5 is the factor difference.

### Stiffness

**Fig. 6:**
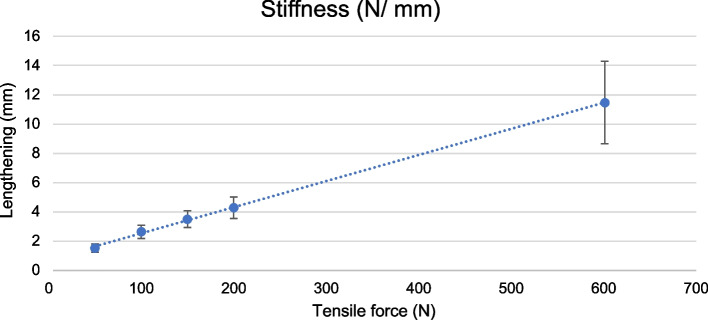
Stiffness of human MCL. Stiffness measured in N/mm of the MCL, showing mean values and SD

When all LTF data on intrasubstance failure are pooled, MCL lengthening is proportional to increasing load, defined as stiffness of MCL (Fig. 6). Stiffness of MCL after completion of puncturing was 52.4 N/mm.

## Discussion

The main finding of this study was that the novel instrument produced a repeatable increase in MCL length in human cadavers. The lengthening was comparable for all rows, demonstrating consistent and repeatable increments, which is an important factor in ligament balancing in TKA. A technique that more often can produce consistent and repeatable balancing results is beneficial as ligament balancing is a prerequisite for good function and survival [2–4]. Furthermore, load-to-failure after puncturing five rows demonstrated tensile strength and stiffness of the MCL ligaments similar to findings in previous studies [9, 10]. The use of the instrument therefore does not seem to diminish the structural properties of or show any detrimental effect on the MCL in an unacceptable manner.

MCL width varies between individuals [11], but the width is related to the number of fibres. With our instrument, a narrower MCL will be punctured fewer times per row than a wide MCL. The gradual lengthening achieved by using the instrument in a sequential fashion is due to two key factors: 1) the tensile strength of the ligament is linear until breaking point [9], and 2) the puncturing grid secures an even distribution of punctures and ensures that the relationship between the number of punctured fibres and intact fibres remain the same. This makes use of the instrument generalizable between patients. The linear equation in human tissue (lengthening per row y = 0.068x – 0.009) is easy to use in the clinical setting as it states that total lengthening is approximately 0.07 mm per row punctured if the constant (-0.009) is ignored. Furthermore, the results show that specimens with an initial greater lengthening for puncturing through row 1 continued to have greater lengthening for subsequent punctured rows. The opposite is also true for specimens showing initial short lengthening for puncturing through row 1 continued to have short lengthening for subsequent punctured rows (Fig. 3). This is associated to MCL width at joint line (*p* = 0.003) and must be kept in mind when puncturing the MCL. One should also be aware that narrow MCLs have a lower LTF.

The total lengthening of the MCL was short, at 0.33 ± 0.20 mm in human tissue. In TKA surgery the joint gap is typically judged subjectively [2], but ligament lengthening of 1–2 mm is often required [3, 8, 12, 13] as this is the minimum that conventional instruments can distinguish and these are the minimum increments for tibial inserts. Koh et al. [14] have previously shown that the pie crusting technique can produce progressive lengthening up to 6 mm in humans with 200 N tensile force. We used 10 N tensile force as our intention was to remove the slack in the ligament without testing the elastic properties. Previous studies of ligament laxity [2, 15] have used a standardized moment of 12 Nm. It is difficult to equate this to tensile force, but Bendjaballah et al. [16] have shown that 12 Nm of valgus strain on the knee produced approximately 170 N of tensile force in the MCL. A systematic review [17] states that the normal tensile force of the MCL during the gait cycle ranges between 20 and 150 N. The normal tensile force during gait is therefore potentially considerably higher than that tested in this study and will influence elastic properties of the MCL. In the present study (Fig. 5), a tensile force of 170 N during LTF produced a mean lengthening of 3.8 ± 0.65 mm. A higher tensile force would therefore produce greater lengthening during the puncturing procedure, and we might have underestimated lengthening compared to Koh et al. [14]. Since the tensile strength of the MCL is linear [9], we can assume that the mean lengthening at 170 N divided by the mean lengthening at 10 N will give us a factor (11.5) by which lengthening per row would have increased if we had performed our testing at 170 N throughout. With this assumption, lengthening per row would have been approximately 0.8 mm. This is clinically relevant lengthening and puncturing of 1–3 rows could produce correct ligament lengthening, measured by spatulas [2], navigated surgery [18] or/ and a ligament tensor (e.g. DePuy CAS Ligament Tensor). On the other hand, tensile forces approaching 200 N are close to the maximum tensile strength of some MCL ligaments and could be detrimental [9].

The main limitations of this biomechanical study were 1) the use of fresh frozen cadaveric tissue, 2) differences between experimental setup and force vectors compared to real life, and 3) the lack of evaluation of other supporting structures around the knee and how this affects gap balancing acutely and in the long term. Furthermore, some might argue that ligament balancing is becoming redundant, as anatomic, kinematic and patient-specific alignment techniques and computer navigation are increasing in popularity. However, studies to date have not shown conclusive evidence that new alignment techniques are superior [19, 20], and mechanical alignment should therefore still be the gold standard. Further, the need for ligament balancing should not be reduced with kinematic or patient-specific strategies. Medial osteoarthritis (60–80%) will still result in MCL contracture due to a non-physiological varus if length is not maintained due to osteophytes or other compensatory mechanisms. Whichever alignment strategy is used, non-physiological contractures should be corrected. Furthermore, computer navigated surgery with haptic robots and tensor devices in TKA can effectively guide the surgeon to produce accurate bone cuts and measure soft tissue tension, but a surgical technique for ligament balancing is lacking. Our instrument could be combined with navigated surgery to enable accurate and objective ligament balancing. Our method of standardizing lengthening procedures could also be studied in other tissues such as lateral structures in valgus knees, tendon contractures, carpal tunnel syndrome or Achilles tendons, as it produces meshing of the structures.

Another disadvantage of the instrument is its lack of differentiation in tightness of the MCL in flexion and extension. Furthermore, we have only studied MCL lengthening in isolation, which does not reveal how ligament lengthening correlates to clinical gap increase. Future research should examine the association between ligament lengthening and gap balancing [5, 14, 21].

We only observed pull-out from insertion on the femoral side. The tibia was kept intact, but the femur was cut as a bone block to be fixed in the clamp of the testing machine. This procedure may have reduced the strength of the femoral insertion, leading to femoral pull-out. Furthermore, ligaments failed through intrasubstance tear failed at 601 ± 165 N which is consistent with the study of Robinson et al. [9] and demonstrate that ligament lengthening was achieved seemingly without significantly weakening the MCL. However structural properties were not compared to the contralateral knee.

Due to strict MDR regulations we are planning future clinical studies to address the limitations and examine the effects of the instrument in vivo for TKA patients.

## Conclusions

Use of the instrument resulted in linear and repeatable lengthening of MCLs in human cadaveric specimens. This could in turn more often produce intentional gaps and ligament balancing, which are important for TKA outcome.

## Data Availability

Data will be made available on reasonable request to the corresponding author.

## Abbreviations

BMI: Body mass index
CIRRO: Center for Implant and Related Research Oslo
Fig.: Figure
Hz: Hertz
kN: Kilonewtons
LTF: Load-to-failure
MCL: Medial collateral ligament
mm: Millimeters
min: Minutes
N: Newtons
Nm: Newton metres
Eng: OUH; Norw: OUS: Oslo University Hospital
REK: Regional Ethical Committee
SD: Standard deviation
TKA: Total knee arthroplasty
